# Association of American Medical Editors

**Published:** 1883-04-20

**Authors:** 


### *ASSOCIATION OF AMERICAN MEDICAL EDITORS.*

The Editors' Association will meet the present year at Cleveland, Ohio—the same place and time of the American Medical Association. It has been arranged for the sessions to meet between the afternoon meetings of the Sections and evening entertainments.

The President of the Association, Dr. N. S. Davis, will address the members on Tuesday evening upon the "*Present Status and Tendencies of the Medical Profession and Journalism.*"

Dr. H. O. Marcy will make an address on Wednesday evening following upon "*Journalism* devoted to the Protection and Concentration of Medical and Surgical Science in Special Departments."

Dr. Stone, of St. Paul, and Dr. Octerlony are also expected to read papers before the body. It is probable that other parties will read papers at the Association.

